# Therapeutic response to four artemisinin-based combination therapies in Angola, 2021

**DOI:** 10.1128/aac.01525-23

**Published:** 2024-02-29

**Authors:** Pedro Rafael Dimbu, Sarah Labuda, Carolina Miguel Ferreira, Felismina Caquece, Kialanda André, Garcia Pembele, Dilunvuidi Pode, Maria Florinda João, Venceslau Mambi Pelenda, Benjamin Nieto Andrade, Breanna Horton, Culzean Kennedy, Samaly S. Svigel, Zhiyong Zhou, Joana F. M. Morais, Joana do Rosário, Filomeno Fortes, José Franco Martins, Mateusz M. Plucinski

**Affiliations:** 1 National Malaria Control Program, Ministry of Health, Luanda, Angola; 2 United States President’s Malaria Initiative, United States Centers for Disease Control and Prevention, Luanda, Angola; 3 PSI Angola, Luanda, Angola; 4 Field Epidemiology Training Program, Ministry of Health, Luanda, Angola; 5 National Institute of Health Research, Ministry of Health, Luanda, Angola; 6 United States Centers for Disease Control and Prevention, Atlanta, Georgia, USA; 7 United States President’s Malaria Initiative, USAID, Luanda, Angola; 8 Institute of Hygiene and Tropical Medicine, Nova University of Lisbon, Lisbon, Portugal; 9 United States President’s Malaria Initiative, Malaria Branch, United States Centers for Disease Control and Prevention, Atlanta, Georgia, USA; The Children's Hospital of Philadelphia, Philadelphia, Pennsylvania, USA

**Keywords:** falciparum, resistance, TES

## Abstract

Monitoring antimalarial efficacy is important to detect the emergence of parasite drug resistance. Angola conducts *in vivo* therapeutic efficacy studies (TESs) every 2 years in its fixed sentinel sites in Benguela, Lunda Sul, and Zaire provinces. Children with uncomplicated *Plasmodium falciparum* malaria were treated with artemether-lumefantrine (AL), artesunate-amodiaquine (ASAQ), dihydroartemisinin-piperaquine (DP), or artesunate-pyronaridine (ASPY) and followed for 28 (AL and ASAQ) or 42 days (DP and ASPY) to assess clinical and parasitological response to treatment. Two drugs were sequentially assessed in each site in February–July 2021. The primary indicator was the Kaplan-Meier estimate of the PCR-corrected efficacy at the end of the follow-up period. A total of 622 patients were enrolled in the study and 590 (95%) participants reached a study endpoint. By day 3, ≥98% of participants were slide-negative in all study sites and arms. After PCR correction, day 28 AL efficacy was 88.0% (95% CI: 82%–95%) in Zaire and 94.7% (95% CI: 90%–99%) in Lunda Sul. For ASAQ, day 28 efficacy was 92.0% (95% CI: 87%–98%) in Zaire and 100% in Lunda Sul. Corrected day 42 efficacy was 99.6% (95% CI: 99%–100%) for ASPY and 98.3% (95% CI: 96%–100%) for DP in Benguela. High day 3 clearance rates suggest no clinical evidence of artemisinin resistance. This was the fourth of five rounds of TES in Angola showing a corrected AL efficacy <90% in a site. For Zaire, AL has had an efficacy <90% in 2013, 2015, and 2021. ASAQ, DP, and ASPY are appropriate choices as artemisinin-based combination therapies in Angola.

## INTRODUCTION

Artemisinin-based combination therapies (ACTs) were introduced in malaria-endemic Sub-Saharan Africa in the mid 2000s, replacing the failing antimalarials chloroquine and sulfadoxine-pyrimethamine (1). The supply of ACTs, driven by large investments from donors including the U.S. President’s Malaria Initiative (PMI) and the Global Fund to Fight AIDS, Tuberculosis and Malaria (GFATM), has expanded rapidly since then, and ACTs have become the most frequently prescribed antimalarials in malaria-endemic Sub-Saharan Africa (2).

Although six ACTs are recommended for use by the World Health Organization (WHO) (3, 4), the primary ACT used in Sub-Saharan Africa is artemether-lumefantrine (AL). This ACT alone accounts for 85% of PMI and GFATM procurements (5). Reasons for lower use of the other ACTs include side effect profile [artesunate-amodiaquine (ASAQ) and artesunate-mefloquine], overlap with drugs used for chemoprevention (ASAQ and artesunate-sulfadoxine-pyrimethamine), and high cost [dihydroartemisinin-piperaquine (DP) and artesunate-pyronaridine (ASPY)].

Following the introduction of ACTs in Sub-Saharan Africa, the region largely enjoyed a period of stable, high ACT efficacy (1). However, recent therapeutic efficacy monitoring has confirmed several foci of emergent partial artemisinin resistance in East Africa, including in Uganda (6), Rwanda (7), and Tanzania. Independently, therapeutic efficacy trials have reported AL efficacies below the key 90% WHO threshold in dispersed sites across the continent, including in Burkina Faso (8), the Democratic Republic of Congo (9), Angola (10–12), Kenya (13), and Tanzania.

Angola was one of the first African countries to report low AL efficacy, as part of its biennial therapeutic efficacy monitoring surveillance system in fixed sentinel sites (10–12, 14). These surveys have identified AL below 90% in 2013 and 2015 (Zaire Province) (10, 11) and 2019 (Lunda Sul Province) (12) (Table S1). In contrast, efficacy of Angola’s two other first-line ACTs, ASAQ and DP, has remained above 90% across all years in all sites (10–12, 14).

Here, we report on the results of the 2021 round of therapeutic efficacy trials in Angola from the fixed sentinel sites in Benguela, Zaire, and Lunda Sul Province. In addition to the three ACTs used in Angola (AL, ASAQ, and DP), the 2021 round also included an arm trialing ASPY, as this is an ACT being considered for use in Angola.

## MATERIALS AND METHODS

### Study design

We implemented a six-arm clinical outcomes trial using an adapted version of the WHO *in vivo* protocol for antimalarial resistance monitoring (15). AL and ASAQ were assessed in Lunda Sul and Zaire, and DP and ASPY were evaluated in Benguela according to the National Malaria Control Program monitoring strategy. We adhered to global best practices for reporting of antimalarial efficacy data (16–18).

### Study population

We enrolled children with uncomplicated *Plasmodium falciparum* monoinfection and history of fever or axillary temperature ≥37.5°C. To account for differences in transmission intensity between provinces, participants were eligible if parasite density measured through microscopy was 1,000–100,000 parasites/µL in low–moderate transmission Benguela, and 2,000–200,000 parasites/µL in moderate–high transmission Lunda Sul and moderate–very high transmission Zaire. Similarly, the age inclusion criterion was 6–143 months in Benguela and 6–59 months in Lunda Sul and Zaire. Patients with danger signs, known allergy or hypersensitivity to study drugs, with antimalarial use in the preceding 2 weeks, hemoglobin ≤8 g/dL, or who were unavailable for the full duration of follow-up were excluded. Participants were recruited from outpatients attending urban health facilities in the capital cities of each province. The target sample size was 73 patients reaching a study endpoint per arm, and a minimum of 100 patients were enrolled per arm to account for loss to follow-up.

### Data collection

The study was conducted between February and July 2021. Study participants were treated with AL, ASAQ, DP, or ASPY for 3 days. Drugs were assessed sequentially in study sites. All drugs were from manufacturers with WHO pre-qualification, and manufacturer-recommended weight bands were used for participant dosing. Treatment was directly observed for all drug doses except for the evening doses for AL. Guardians of participants in the AL arms were given the evening doses to administer at home and were contacted by telephone in the evening as a reminder; guardians were asked to confirm that drug administration had occurred the following morning in the clinic. All AL doses given in the health facility were administered together with either yogurt or milk, and guardians of participants were provided a yogurt or milk pack each of the first 3 days to give concomitantly with the evening dose.

Study nurses or clinicians performed a clinical assessment at enrollment and on all follow-up days (days 1, 2, 3, 7, 14, 21, and 28). Participants in the DP and ASPY arms were additionally followed on days 35 and 42. Slide microscopy and collection of blood on Whatman 903 filter paper were performed on enrollment and all days except for day 1. Slides were independently read by two microscopists and any discordance >25% triggered a third reading. Slides were prepared and read according to standard WHO guidelines (15). Filter paper was dried overnight and stored at room temperature with individual desiccant packs. Hemoglobin concentration was measured using HemoCue machines (Hemocue, Ängelholm, Sweden) at enrollment and every 14 days during follow-up. Rescue treatment was intravenous artesunate for participants developing severe malaria or an alternate ACT for treatment failures without severe illness.

### Molecular analysis

Paired filter paper samples from participants with recurrent parasitemia, defined as presence of any *P. falciparum* asexual parasites on slide microscopy on or after day 7, were stored at room temperature in individual plastic bags with desiccant, and then sent to Centers for Disease Control and Prevention (CDC) laboratories in Atlanta, USA, for molecular correction. Four 3.0 mm punches were taken from each dried blood spot sample and placed into a 1.5 mL tube. DNA was extracted using the QIAamp DNA Mini Kit (Qiagen, Valencia, California) according to manufacturer specifications and standardized procedures. A DNA eluent solution of 200 µL was stored at −20°C. Prior to genotyping, presence of *P. falciparum* DNA was confirmed with photo-induced electron transfer-PCR (19). The fragment lengths of seven neutral microsatellites (313, 383, TA1, POLYA, PFPK2, 2490, and TA109) were measured using capillary electrophoresis as previously described (20, 21). The CDC Bayesian algorithm for molecular correction was used to assign a posterior probability of recrudescence for each case of recurrent parasitemia (22).

### Statistical analysis

Data were collected on paper forms that were digitized the same day using double-data entry. Study endpoints included treatment success (formally denoted as adequate clinical and parasitological response), early treatment failure, and late treatment failure, using the canonical definitions from the WHO protocol (15). The primary outcome was the Kaplan-Meier estimate of the corrected day 28 efficacy for AL and ASAQ, and day 42 efficacy for DP and ASPY. Secondary outcomes included day 3 slide positivity rates; rates of adverse events; the Kaplan-Meier estimate of uncorrected day 28 efficacy for AL and ASAQ, and uncorrected day 42 efficacy for DP and ASPY; and Kaplan-Meier estimates of the corrected and uncorrected day 28 efficacies for DP and ASPY. In the Kaplan-Meier method, cases of loss to follow-up and indeterminate PCR results were censored at last day of follow-up, and the R survival package was used to generate the Kaplan-Meier estimates. Posterior probabilities of recrudescences were incorporated into the Kaplan-Meier estimates as previously described (22). All outcomes were calculated stratifying by study arm.

All data analyses were done using R version 4.1.2 (R Foundation for Statistical Computing, Vienna, Austria). The R script used to generate the data is available at https://github.com/MateuszPlucinski/AngolaTES2021.

## RESULTS

A total of 622 patients were enrolled in the study (Table 1). Between 448 and 1183 children were screened for each arm; the most common reasons for non-eligibility were absence of *P. falciparum* infection (77% of exclusions), *P. falciparum* infection with parasite density outside of required range (12%), or hemoglobin outside of the required range (4%). Median baseline parasite density ranged from 31,324–55,264 parasites/µL by study arm, and median age varied from 2.5 to 2.7 years in Zaire and Lunda Sul and was 6.9–7.0 years in the Benguela arms.

**TABLE 1:** Number of participants screened, enrolled, and finishing follow-up and characteristics at baseline as part of therapeutic efficacy monitoring in Angola, 2021^*a*^

	Benguela		Zaire		Lunda Sul	
	ASPY	DP	AL	ASAQ	AL	ASAQ
Total screened, *N*	1,183	794	448	606	775	868
Total enrolled, *N*	104	105	104	105	104	100
Loss to follow-up, *n* (%)	2 (2)	1 (1)	2 (2)	6 (6)	3 (3)	7 (7)
Exclusion, *n* (%)	2 (2)	0 (0)	4 (4)	2 (2)	1 (1)	2 (2)
Reached study endpoint, *n* (%)	100 (96)	104 (99)	98 (94)	97 (92)	100 (96)	91 (91)
Participant characteristics at baseline						
Median age, years (range)	6.9 (0.5–12)	7 (0.6–12)	2.5 (0.5–5)	2.5 (0.7–5)	2.6 (0.5–5)	2.7 (0.6–5)
Median weight, kg (range)	18 (8–52)	18 (7–39)	12 (6–18)	12 (7–18)	12 (7–19)	12 (7–17)
% Female	49%	47%	45%	56%	49%	46%
Median baseline parasite density, p/µL (range)	31,324 (1,011–99,604)	33,863 (1,159–97,916)	45,308 (2,056–190,755)	55,264 (3,469–186,403)	38,377 (3,692–162,624)	51,329 (3,796–178,458)
Median baseline hemoglobin, g/dL (range)	10.6 (8.1–14.2)	10.7 (8.1–14.8)	10.6 (8.1–14.5)	10.4 (8.1–14.1)	10.6 (8.1–14.2)	10.4 (8.1–14)
Months of enrollment	Mar’21–May’21	May’21–Jul’21	Feb’21–Mar’21	Mar’21–Jun’21	Feb’21–Apr’21	Mar’21–Jun’21

^*a*^ ASPY, artesunate-pyronaridine; DP, dihydroartemisinin-piperaquine; AL, artemether-lumefantrine; ASAQ, artesunate-amodiaquine.

Combined loss to follow-up and exclusion rates were below 10% for all study arms, and the number of participants reaching a study endpoint ranged from 91 to 104 per arm (Table 1). Reasons for exclusion included non-falciparum infection during follow-up (*n* = 5), development of danger signs without parasitemia (*n* = 3), development of danger signs within 24 hours of enrollment (*n* = 2), and use of other antimalarials (*n* = 1).

Day 2 negativity ranged from 74% to 94%, while day 3 negativity was above 98% for all study sites and arms (Table 2). Rates of reported side effects were low (<1%) across all study sites and arms (Table S2).

**TABLE 2:** Proportion of slides negative for asexual malaria parasites on days 2 and 3 following antimalarial treatment, therapeutic efficacy monitoring in Angola, 2021^*a*^

	Benguela				Zaire				Lunda Sul			
	ASPY		DP		AL		ASAQ		AL		ASAQ	
	*n*/*N*	% (95% CI)	*n*/*N*	% (95% CI)	*n*/*N*	% (95% CI)	*n*/*N*	% (95% CI)	*n*/*N*	% (95% CI)	*n*/*N*	% (95% CI)
Day 2 slide negativity	98/104	94 (87–98)	88/104	85 (76–91)	89/104	86 (77–91)	92/101	91 (83–96)	77/104	74 (64–82)	75/98	77 (67–84)
Day 3 slide negativity	104/104	100 (96–100)	103/105	98 (93–100)	102/102	100 (95–100)	101/101	100 (95–100)	101/103	98 (92–100)	99/99	100 (95–100)

^*a*^ ASPY, artesunate-pyronaridine; DP, dihydroartemisinin-piperaquine; AL, artemether-lumefantrine; ASAQ, artesunate-amodiaquine; CI, confidence interval.

Among participants reaching a study endpoint, there were a total of four early treatment failures and 71 cases of recurrent parasitemia after day 7 (late treatment failures) (Table 3). Of the 71 late treatment failures, one sample from the Benguela ASPY arm was classified as indeterminate due to a missing sample, 24 infections were classified as recrudescences, and 46 infections were classified as new infections. Most posterior probabilities of recrudescence (58/70, 83%) were either <0.1 or >0.9, suggesting high statistical confidence in the classification (Table S3).

**TABLE 3:** Treatment outcomes for participants finishing follow-up as part of therapeutic efficacy monitoring in Angola, 2021^*a*^^,*b*^

	Benguela		Zaire		Lunda Sul	
	ASPY	DP	AL	ASAQ	AL	ASAQ
	*n* (%)					
	*N* = 100	*N* = 104	*N* = 98	*N* = 97	*N* = 100	*N* = 91
Treatment failure	14 (14)	6 (6)	23 (23)	19 (20)	13 (13)	0
Early treatment failure	0	0	1 (1)	3 (3)	0	0
Late treatment failure	14 (14)	6 (6)	22 (22)	16 (16)	13 (13)	0
Recrudescence	0	2 (2)	10 (10)	6 (6)	6 (6)	0
Day 7	0	1 (1)	0	0	0	0
Day 14	0	0	3 (3)	0	3 (3)	0
Day 21	0	0	5 (5)	4 (4)	2 (2)	0
Day 28	0	0	2 (2)	1 (1)	1 (1)	0
Day 35	0	1 (1)				
Day 42	0	0				
New infection	13 (13)	4 (4)	12 (12)	10 (10)	7 (7)	0
Day 7	0	0	0	0	0	0
Day 14	0	0	2 (2)	1 (1)	2 (2)	0
Day 21	1 (1)	0	5 (5)	3 (3)	3 (3)	0
Day 28	3 (3)	2 (2)	5 (5)	6 (6)	2 (2)	0
Day 35	4 (4)	0				
Day 42	5 (5)	2 (2)				
Adequate clinical and parasitological response	86 (86)	98 (94)	75 (77)	78 (80)	87 (87)	91 (100)

^*a*^ One late treatment failure from the Benguela ASPY arm was indeterminate for PCR correction (sample missing).

^*b*^ ASPY, artesunate-pyronaridine; DP, dihydroartemisinin-piperaquine; AL, artemether-lumefantrine; ASAQ, artesunate-amodiaquine.

Uncorrected day 28 efficacies ranged from 77.1% (95% CI: 69%–86%) in Zaire for AL to 100% for ASAQ in Lunda Sul (Table 4). Day 42 uncorrected efficacy was 87.2% (95% CI: 81%–94%) for ASPY and 94.2% (95% CI: 90%–99%) for DP, both in Benguela. After PCR correction, AL efficacy was 88.0% (95% CI: 82%–95%) in Zaire and 94.7% (95% CI: 90%–99%) in Lunda Sul. For ASAQ, efficacy was 92.0% (95% CI: 87%–98%) in Zaire and 100% in Lunda Sul. Corrected day 42 efficacy was 99.6% (95% CI: 99%–100%) for ASPY and 98.3% (95% CI: 96%–100%) for DP.

**TABLE 4:** Efficacy of first-line antimalarials in three therapeutic efficacy monitoring sites in Angola in 2021^*b*^

	Benguela		Zaire		Lunda Sul	
	ASPY	DP	AL	ASAQ	AL	ASAQ
	% (95% CI)					
Uncorrected Kaplan-Meier estimate						
Day 28	96.1 (93–100)	97.1 (94–100)	77.1 (69–86)	82.9 (76–91)	87.3 (81–94)	100^*a*^
Day 42	87.2 (81–94)	94.2 (90–99)				
PCR-corrected Kaplan-Meier estimate						
Day 28	99.7 (99–100)	99 (97–100)	88 (82–95)	91 (85–97)	94.4 (90–99)	100^*a*^
Day 42	99.6 (99–100)	98.3 (96–100)				

^*a*^ Confidence intervals are undefined.

^*b*^ ASPY, artesunate-pyronaridine; DP, dihydroartemisinin-piperaquine; AL, artemether-lumefantrine; ASAQ, artesunate-amodiaquine.

## DISCUSSION

This is the fourth of five rounds of TES in Angola showing a corrected AL efficacy below 90% in a site. For Zaire, AL has had an efficacy below 90% in 2013, 2015, and 2021. These notable Zaire AL estimates are both close to each other (88.0%–89.6%) but also close to the 90% WHO threshold. The uncorrected efficacies for AL in Zaire have also been relatively stable over all five rounds: 77.4% in 2013, 76.0% in 2015, 92.8% in 2017, 84.2% in 2019, and 77.1% in 2021. Given the stability of both the uncorrected and corrected results, it is likely that these results reflect the true background susceptibility of parasites in this region to lumefantrine.

In contrast to the AL findings, in this study, ASAQ, DP, and ASPY all had corrected efficacies above 90% and the results suggest that they are appropriate choices for ACTs in Angola. To date, no drug other than AL has had a corrected efficacy below 90% in any round of therapeutic efficacy monitoring in Angola. As in previous rounds, day 3 slide-negativity rates were high across all study sites and arms, continued evidence of high artemisinin susceptibility in Angolan parasites.

This was the first study assessing ASPY efficacy in Angola. Although the uncorrected day 42 efficacy for the ASPY arm was 87.2%, all 13 cases of late treatment failure were ultimately classified as new infections. Notably, nine (70%) of these cases were observed on day 35 or day 42. The half-life of pyronaridine has been estimated to be 10–13 days (23). This is very similar to the estimated half-life of 9–12 days for desethylamodiaquine (24, 25), the long-lasting metabolite of amodiaquine, and much shorter than the estimated half-life of piperaquine of 23 days (26). As such, based on partner drug half-lives, a 28-day follow-up might be more appropriate for ASPY. If the trend of new infections predominantly occurring after day 28 in ASPY trials is confirmed in other settings, the global antimalarial resistance surveillance community might consider shortening the follow-up period for ASPY to 28 days.

In late 2021, the WHO revised its guidelines for genotyping parasites for molecular correction of antimalarial resistance trials (27). This was in response to mounting evidence that the 2007 guidelines were insufficiently sensitive in identifying recrudescences (28, 29). The new guidelines recommended use of *msp1*/*msp2* and a microsatellite for all studies, in place of earlier protocols like *msp1*/*msp2*/*glurp* (widely used) and panels of microsatellites (used primarily by CDC). The timing of the guideline release meant that molecular correction for this 2021 TES was still performed using the older panel of seven neutral microsatellites. When regarding the distribution of posterior probabilities of recrudescence, most cases of recurrent cases of parasitemia classified as recrudescences had substantial statistical support to infer recrudescence (posterior probability >0.9). Notably, 22 of 24 (92%) recurrent cases of parasitemia classified as recrudescences by the Bayesian algorithm had matches at ≥4/7 loci. Conversely, all 46 new infections had matches at <4/7 loci. This very closely confirms earlier predictions from two independent modeling approaches suggesting that the 4/7 threshold is the most appropriate threshold for differentiating recrudescences from new infections using seven neutral microsatellites (30, 31). Moreover, because previous rounds of TES monitoring in Angola also used the same panel and Bayesian analysis approach, corrected efficacies can be directly compared between this study and studies from previous years.

In addition to uncertainties related to the molecular correction, there are other limitations inherent to the study design. Sequential enrollment of patients for study arms means that drug efficacies should not be directly compared as patients were exposed to different risk of acquiring new infections during follow-up. Lack of direct observation of the evening doses of AL poses a risk of non-adherence and underdosing; however, procedures implemented to minimize this risk, including telephoning patients with reminders and verification of used blister packs, mean that systematic underdosing was unlikely. Molecular markers of detection, useful in complementing TES results, will be reported separately.

The stability of the long-term trends in the period 2013–2021 for AL, ASAQ, and DP augments the robustness of the findings, and suggests that ASAQ and DP may be more appropriate ACTs than AL in Angola. Although just the first data point in Angola, the high efficacy of ASPY suggests it may also be an appropriate ACT for the country. Continued monitoring should be conducted to confirm these conclusions.

## Data Availability

The full clinical data set has been uploaded to WorldWide Antimalarial Resistance Network and WHO repositories. Full genotyping data are available in Table S3 and the full clinical data set is available in Table S4.

## SUPPLEMENTAL MATERIAL

Supplemental Tables S1 and S2 aac.01525-23-s0001.docx Supplemental tables.

Supplemental Table S3 aac.01525-23-s0002.xlsx Genotyping database.

Supplemental Table S4 aac.01525-23-s0003.xlsx Full clinical database.

ASM does not own the copyrights to Supplemental Material that may be linked to, or accessed through, an article. The authors have granted ASM a non-exclusive, world-wide license to publish the Supplemental Material files. Please contact the corresponding author directly for reuse.
