# Supplementary material for: Therapeutic response to four artemisinin-based combination therapies in Angola, 2021
Source: Antimicrob Agents Chemother. 2024 Feb 29;68(4):e01525-23. doi: 10.1128/aac.01525-23 (PMC10989004; doi:10.1128/aac.01525-23)
Supplement: Supplemental Tables S1 and S2 — Supplemental tables. [file aac.01525-23-s0001.docx]

| **Supplemental Table S1**. Kaplan-Meier estimates of Day 28 (AL and ASAQ) and Day 42 (DP and ASPY) PCR-corrected efficacies in Angolan sentinel sites since start of therapeutic efficacy monitoring of artemisinin-based combination therapies | | | | | | | | | | | | | | | | |
| --- | --- | --- | --- | --- | --- | --- | --- | --- | --- | --- | --- | --- | --- | --- | --- | --- |
|  | Zaire | | | |  | Benguela | | | |  | Lunda Sul | | |  | Uíge* | |
| Year | AL | ASAQ | DP | ASPY |  | AL | ASAQ | DP | ASPY |  | AL | ASAQ | DP |  | AL | DP |
| 2013 | 89.6 | - | 100** | - |  | - | - | - | - |  | - | - | - |  | 97.4 | 100** |
| 2015 | 88.1 | - | 98.8 | - |  | 96.3 | 99.9 | - | - |  | - | 100 | 100 |  | - | - |
| 2017 | 95.5 | 93.3 | - | - |  | - | 100 | 100 | - |  | 96.5 | - | 100 |  | - | - |
| 2019 | 92.2 | 95.6 | - | - |  | 98.4 | 100 | - | - |  | 87.6 | 100 | - |  | - | - |
| 2021 | 88.0 | 91.1 | - | - |  | - | - | 98.3 | 99.6 |  | 94.4 | 100 | - |  | - | - |
| 2023 | *** | - | - | *** |  | *** | - | *** | - |  | *** | *** | - |  | - | - |
| Numbers in cells represent % | | | | | | | | | | | | | | | | |
| *discontinued after 2013 | | |  |  |  |  |  |  |  |  |  |  |  |  |  |  |
| **Day 28 only | |  |  |  |  |  |  |  |  |  |  |  |  |  |  |  |
| ***planned |  |  |  |  |  |  |  |  |  |  |  |  |  |  |  |  |
| ASPY: Artesunate pyronaridine; DP: Dihydroartemisinin piperaquine; AL: Artemether lumefantrine; ASAQ: Artesunate amodiaquine | | | | | | | | | | | | | | | | |

| **Supplemental Table S2**. Prevalence of adverse events reported after antimalarial treatment, therapeutic efficacy monitoring in Angola, 2021 | | | | | | |
| --- | --- | --- | --- | --- | --- | --- |
|  | **Benguela** | | **Zaire** | | **Lunda Sul** | |
|  | **ASPY** | **DP** | **AL** | **ASAQ** | **AL** | **ASAQ** |
|  | N=104 | N=105 | N=104 | N=105 | N=104 | N=100 |
| Vomiting, n (%) | 0 (0) | 0 (0) | 0 (0) | 0 (0) | 0 (0) | 0 (0) |
| Diarrhea, n (%) | 0 (0) | 0 (0) | 1 (1) | 0 (0) | 0 (0) | 0 (0) |
| Nausea, n (%) | 0 (0) | 0 (0) | 0 (0) | 0 (0) | 0 (0) | 0 (0) |
| Sweating, n (%) | 0 (0) | 0 (0) | 0 (0) | 0 (0) | 0 (0) | 0 (0) |
| ASPY: Artesunate pyronaridine; DP: Dihydroartemisinin piperaquine; AL: Artemether lumefantrine; ASAQ: Artesunate amodiaquine | | | | | | |
